# Beyond Cystic Fibrosis: Recognising Shwachman‐Diamond Syndrome in the Respiratory Clinic

**DOI:** 10.1002/rcr2.70440

**Published:** 2025-12-10

**Authors:** Freda Yang, Ladina Weitnauer, Imogen Felton, Nicholas J. Simmonds

**Affiliations:** ^1^ National Heart and Lung Institute, Imperial College London London UK; ^2^ Guy's ‐ Brompton Asthma Network, Royal Brompton Hospital, Guy's and St Thomas' NHS Foundation Trust London UK; ^3^ Adult Cystic Fibrosis Centre, Royal Brompton Hospital, Guy's and St Thomas' NHS Foundation Trust London UK

**Keywords:** CFTR, cystic fibrosis, SDBS, Shwachman‐Diamond Syndrome

## Abstract

Cystic fibrosis (CF) and Shwachman‐Diamond Syndrome (SDS) share overlapping features, including recurrent respiratory infections and pancreatic insufficiency, which can complicate diagnosis. We report a case of a six‐year‐old girl who presented with productive cough, steatorrhea and recurrent infections. Initial evaluation showed abnormal liver enzymes, elevated immunoreactive trypsin, neutrophil dysfunction and skeletal anomalies. Sweat test was equivocal and CFTR genetic panel was negative. Further genomic analysis identified compound heterozygous mutations in the SBDS gene: c.258+2T>C (known pathogenic variant) and c.284T>A (novel variant), confirming SDS. Over a 42‐year follow‐up, she experienced intermittent neutropenia, recurrent respiratory infections and pregnancy‐related complications. This case emphasises the importance of considering SDS in CF‐like presentations with atypical features and equivocal CF testing. Milder SDS phenotypes can survive into adulthood. Further work is needed to refine genotype–phenotype correlations and guide long‐term management.

## Graphical abstract

Our case shows how SDS can mimic CF (borderline sweat electrolytes, recurrent lower respiratory infections, pancreatic insufficiency) but diverge in trajectory and management. Over 42 years, our patient demonstrated: (1) recurrent infections, (2) persistent pancreatic insufficiency and (3) intermittent neutropenia. Definitive SDS confirmation came decades later with SBDS c.258+2T>C in trans with a novel missense variant (c.284T>A), which is a point of genuine novelty for the literature and directly relevant to modern diagnostic pathways when CFTR testing is negative or equivocal.

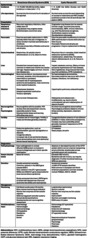

## 1 Introduction

Cystic Fibrosis (CF) is a well‐known autosomal recessive disorder primarily affecting the respiratory and gastrointestinal systems, characterised by recurrent respiratory infections, bronchiectasis and exocrine pancreatic insufficiency [1]. Shwachman‐Diamond Syndrome (SDS) is an extremely rare condition (1 in 75,000–100,000 live births) that can mimic CF during childhood due to overlapping features such as recurrent infections and pancreatic dysfunction [2]. Given this overlap, clinicians should consider SDS in CF‐like presentations, particularly when atypical features, such as cytopenia or skeletal anomalies, are present. We describe a patient diagnosed with SDS in childhood and followed for 42 years, highlighting key differences in evaluation, management and prognosis.

## 2 Case Report

A six‐year‐old girl presented with a one‐week history of productive cough and chronic steatorrhea. She was born at 41 weeks and was ‘smaller than her peers’ throughout childhood. Past history included sinusitis, eczema and recurrent respiratory, urinary tract and skin infections. Family history featured respiratory illnesses (pneumonia and tuberculosis). Her 29‐year‐old mother had adult‐onset asthma, bronchiectasis, recurrent nasal polyps, and mixed hearing loss. Examination identified bony anomalies.

Laboratory investigations showed elevated liver transaminases (GGT, ALT, AST) and serum immunoreactive trypsin, which suggested exocrine pancreatic insufficiency. Sputum culture grew *Haemophilus influenzae* . Immunological testing showed impaired neutrophil locomotion and chemotaxis, with preserved monocyte function.

Sweat testing showed borderline sweat sodium concentrations (43 and 48 mEq/L), the diagnostic gold standard for CF at that time. Ciliary function was normal. A presumptive diagnosis of SDS was made based on clinical presentation and equivocal sweat results. CF genetic testing, approximately 12 years later, found no identifiable cystic fibrosis transmembrane conductance regulator (CFTR) variants. Comprehensive SDS genetic analysis (bi‐directional Sanger sequencing in all five exons of the Schwachman‐Bodian‐Diamond syndrome [SBDS] gene) identified a pathogenic variant (c.258+2T>C) and in trans novel missense variant (c.284T>A, p.Val95Glu). This codon has been previously implicated in SDS, with a different amino acid substitution at the same position (c.284T>G; p.Val95Gly) reported by Scalais et al. [3], which supports the pathogenicity of our variant and provides molecular confirmation of SDS 29 years after the initial clinical diagnosis.

In adulthood, she experienced a miscarriage at 25 weeks and pre‐eclampsia in two subsequent pregnancies. Aged 48, she has intermittent neutropenia. Liver transaminases are within the normal range, but she remains pancreatic insufficient. Despite frequent lower respiratory infections, spirometry is normal. Chest CT shows bronchial wall thickening and mucous plugging without bronchiectasis.

## 3 Discussion

SDS and CF overlap in early life, most notably with pancreatic insufficiency and recurrent infections, but skeletal anomalies, cytopenia and bone marrow dysfunction are characteristics of SDS [2]. SDS should be considered when CF investigations are negative or equivocal, particularly if extensive CFTR analysis is inconclusive.

CF results from CFTR protein dysfunction leading to dehydrated secretions, ductal obstruction and infection [1]. SDS is due to biallelic mutations in SBDS, a gene involved in ribosome assembly. Infection risk in SDS is driven in part by neutrophil dysfunction, particularly impaired chemotaxis [4]. Interestingly, both SBDS and CFTR are located on chromosome 7.

Pancreatic disease occurs in both conditions but often diverges over time. In SDS, > 90% have early exocrine dysfunction from fatty infiltration and acinar atrophy, but function may improve with age. In CF, this results from obstruction of pancreatic ducts, which leads to progressive fibrosis and pancreatic insufficiency (sometimes acute recurrent pancreatitis is the main feature) [1].

Liver involvement also differs. In SDS, elevated transaminases are common in childhood and often resolve, though cholestasis and cirrhosis can occur. In CF, liver issues include fatty infiltration or biliary cirrhosis [1].

Skeletal and haematological findings distinguish SDS from CF. Metaphyseal dysplasia, delayed epiphyseal ossification, and low‐turnover osteoporosis may occur in SDS [2]. Bone‐marrow failure manifests as intermittent or persistent cytopenia, with a significant risk (10%–36%) of transformation into myelodysplastic syndrome and acute myeloid leukaemia [5]. This mostly accounts for the reduced life expectancy (age 38–41). Recurrent miscarriages occur more frequently in SDS, though live birth rates remain similar to the general population [6]. Additional SDS features are summarised in Table 1.

**TABLE 1:** Summary of Shwachman‐Diamond Syndrome (SDS) versus Cystic Fibrosis (CF).

	Shwachman‐Diamond Syndrome (SDS)	Cystic Fibrosis (CF)
Epidemiology		
Incidence	1 in 75,000–100,000 live births, higher incidence in males (ratio 1.7:1)	1 in 2500 live births (Europe and North America)
Life expectancy	Survival into the third and fourth decades of life reported	Survival varies across the world depending on access to treatments and MDT (median predicted survival in the UK and US was > 60 years in 2023)
Presentation		
Respiratory disease & Infections	Recurrent respiratory infections, often milder than CF Lung disease may progress slowly. Bronchiectasis uncommon early	Progressive lung disease (can be severe) Chronic cough, recurrent pneumonia, bronchiectasis, ABPA, chronic infections, for example, with *Pseudomonas aeruginosa* , *Burkholderia cepacia* complex, Mycobacterium abscesses
Pancreatic	Exocrine pancreatic dysfunction in > 90%, typically within first year of diagnosis. In some, function improves spontaneously in childhood Pancreas shows fatty infiltration and acinar cell atrophy	Exocrine pancreatic insufficiency is common (90% patients) due to ductal obstruction by thick mucus Pancreatic fibrosis and atrophy are progression. Enzyme replacement is lifelong
Gastrointestinal	Steatorrhea, fat‐soluble vitamin deficiencies (A, D, E, K), failure to thrive	Meconium ileus in neonates; distal intestinal obstruction syndrome (DIOS); malabsorption, steatorrhea. Fat‐soluble vitamin deficiencies (A, D, E, K). Failure to thrive
Liver	Elevated liver transaminases are very common in infants and children but resolve with age. Hepatomegaly common. Chronic liver disease rare	Hepatic steatosis common. Biliary cirrhosis and portal hypertension (CF liver disease). Risk of gallstones. Drug‐induced liver injury (e.g., from antibiotics, CFTR modulators)
Haematological	Bone marrow failure: neutropenia (most common), anaemia, thrombocytopenia are intermittent or persistent Increased risk of myelodysplastic syndrome and leukaemia, most commonly acute myeloid leukaemia	No haematological abnormalities Secondary anaemia may occur with chronic disease, infections, iron‐deficiency
Skeletal	Metaphyseal dysplasia Delayed epiphyseal ossification Thoracic dystrophy Low‐turnover Osteoporosis	Hypertrophic pulmonary osteoarthropathy Osteopenia and osteoporosis – multifactorial, for example, due to chronic corticosteroid use and malabsorption of vitamin D and K
Neurocognitive and Psychological	Neurocognitive deficits possible. MRI studies show decreased brain volume, especially posterior fossa Developmental delays and learning difficulties may occur	No direct neurocognitive abnormalities Psychological problems related to chronic illness (anxiety, depression, treatment burden)
Fertility	Increased rate of miscarriages. Fertility in males and females generally preserved	Congenital bilateral absence of vas deferens (CBAVD) in males; obstructive azoospermia Females may have subfertility (which generally improves if responsive to CFTR modulators)
Others	Endocrine dysfunction, such as hypothyroidism, glucose dysregulation or diabetes Congenital defects Increased risk of oral and dental disease Visual abnormalities (rod‐cone dystrophy) Sensorineural hearing loss	CF diabetes Nasal polyps, sinusitis
Diagnostics		
Pathophysiology	Exact pathogenesis is unclear Affected SDBS protein is involved in ribosome biogenesis and mitotic spindle stabilisation	Absence or decreased function of the CFTR protein which is an ion channel transporting anions (chloride, bicarbonate) across epithelial apical cell membranes
Sweat chloride levels	Below 60 mmol/L	Elevated (≥ 60 mmol/L) Borderline (30–59 mmol/L), but may be diagnostic if further functional tests (e.g., NPD) are abnormal
Genetics	Usually autosomal recessive Common: variants in the SBDS gene (causes SDS1) Rare: variants in the *EFL1* gene (causes SDS2) and *DNAJC21* gene (responsible for bone marrow failure syndrome) Rarely autosomal dominant: *SRP54* gene (is linked with Shwachman‐Diamond syndrome like (SDSL) and neutropenia)	Autosomal recessive > 2000 CFTR variants identified Most common in European and North American populations: common variants include *F508del*, *G551D*, *G542X*
Management		
Monitoring	Full blood counts every 3–6 months Bone marrow monitoring as indicated Monitor liver enzymes and fat‐soluble vitamins DEXA during growth and after puberty Annual dental examinations Developmental and neurocognitive assessment	Lung function (spirometry) Sputum cultures Monitor liver enzymes and fat‐soluble vitamins HbA1c/OGTT Nutrition and weight monitoring DEXA every 3–5 years
Multidisciplinary Team	Haematology Pulmonologist Gastroenterology Orthopaedics Paediatrics Genetics Dental	Pulmonology (CF specialist) Paediatrics Endocrine (CF diabetes) Gastroenterology CF specialist nurses Respiratory/physical therapists (physiotherapy) Psychology Pharmacy Dietetics

Abbreviations: ABPA, allergic bronchopulmonary aspergillosis; CFTR, cystic fibrosis transmembrane conductance regulator; DEXA, dual‐energy X‐ray absorptiometry; HbA1c, glycated haemoglobin; MDT, multidisciplinary team; MRI, magnetic resonance imaging; NPD, nasal potential difference; OGTT, oral glucose tolerance test; SBDS, Schwachman‐Bodian‐diamond Syndrome.

Diagnosis relies on clinical suspicion supported by genetic analysis [2]. In the Boston Children's SDS Registry (*n* = 419), SBDS mutations were detected in 62%; 4% had rarer implicated genes, 34% had no identifiable genetic cause. In our case, further supporting its likely pathogenic nature, a novel missense variant at a previously reported pathogenic codon was identified (c.284T>A, p.Val95Glu) [3], further supporting its likely pathogenic nature.

Management overlaps, but key differences exist. Both conditions require respiratory care, pancreatic enzyme replacement and attention to bone health and fat‐soluble vitamins. SDS additionally requires haematologic monitoring and surveillance for clonal evolution/malignancy [7].

This case highlights two practical messages:

1. Think beyond CF. Consider SDS in CF‐like presentations with atypical features and equivocal or negative CF diagnostic tests.
2. Milder phenotypes of SDS survive into adulthood. Respiratory physicians should recognise SDS as an important differential for recurrent respiratory infections and pancreatic insufficiency where there is no secure CF diagnosis.

## Author Contributions

Acquisition of patient history: F.Y., L.W. Drafting the manuscript: F.Y. Critical revision of the manuscript: F.Y., L.W., I.F., N.J.S. Guarantor: N.J.S.

## Consent

The authors declare that written informed consent was obtained for the publication of this manuscript and attest that the form used to obtain consent from the patient complies with the journal requirements as outlined in the author guidelines.

## Conflicts of Interest

F.Y. has received honoraria for attending meetings and speaker fees from AstraZeneca and GlaxoSmithKline, and is a member of the BTS asthma specialist advisory group. L.W. has no conflicts of interest; I.F. has received honoraria for educational symposia by Vertex. N.J.S. has received honoraria for advisory boards from Vertex, Boehringer Ingelheim, Chiesi, Gilead, and Menarini. He has also received honoraria for educational activities from Vertex, Chiesi, Gilead, Medison, Teva, and Zambon.

## Data Availability

The data that support the findings of this study are available on request from the corresponding author. The data are not publicly available due to privacy or ethical restrictions.
